# Design and evaluation of an agent-based digital-twin-inspired simulation for metacognition research in higher education

**DOI:** 10.3389/fpsyg.2026.1914901

**Published:** 2026-10-23

**Authors:** Chuanhui Guo

**Affiliations:** Academic Affairs Office, Quanzhou Preschool Education College, Quanzhou, China

**Keywords:** agent-based simulation, AMERT model, digital-twin-inspired modeling, higher education, metacognition, metacognitive resilience

## Abstract

**Background:**

There is a significant gap between sophisticated models of metacognition and empirical testing of these models in educational settings, especially the hierarchical AMERT model. Agent-based simulation frameworks, and their conceptual evolution toward simulation-based digital twins, remain largely underutilized in higher education as virtual laboratories for hypothesis generation, despite their potential to model complex developmental dynamics.

**Objective:**

This study aimed to design and internally evaluate a simulation-based digital twin framework that combines the five-level AMERT model and agent-based architecture to investigate the dynamics of metacognitive development.

**Method:**

Using Design and Development Research (DDR), we developed the framework through theoretical synthesis and subjected its conceptual content to expert review via a modified Delphi method with 12 experts. Student agents were simulated using NetLogo and represented four computational learner archetypes. Instructional scenarios were simulated for 150 ticks and repeated 50 times for each scenario, and internal consistency of the simulation logic was examined using Graph Neural Networks; the outputs remained systematically distinguishable across the predefined computational archetypes (89% classification accuracy).

**Findings:**

The hybrid scenario (instructor feedback, peer feedback, and high task complexity) resulted in the highest weighted AMERT mean (3.86) and a modeled self-transfer rate of 38% (assuming the model’s parameters). Feedback from the model teacher was correlated with a 38% decrease in simulated metacognitive gap. Importantly, simulated interventions were modeled as being uniform, which led to a greater difference in performance between the high- and low-performing agent profiles, a mathematically expected pattern under the threshold-based progression mechanism that aligns with the Matthew Effect concept and generates a hypothesis for empirical exploration in practice. All findings should be considered model-based hypotheses, not observations of real students.

**Conclusion:**

This is one of the first efforts to use simulation-based digital twin modeling to metacognition in higher education, providing a proof-of-concept framework for exploring complex metacognitive theory and proposing hypotheses for empirical testing in the future.

## 1 Introduction

Metacognition, “thinking about thinking,” has emerged as a foundational concept in cognitive psychology and educational sciences over the past three decades. The hierarchical AMERT model organizes this capacity into five progressive levels: self-awareness, self-monitoring, self-evaluation, self-regulation, and self-transfer (Willison et al., 2024). This framework positions metacognitive development as the pinnacle of learning achievement, potentially surpassing even general intelligence. In higher education, metacognition becomes critical as students face complex tasks, ill-structured problems, and information overload that resist superficial cognitive strategies (Samon and Levy, 2017).

Despite theoretical consensus, a troubling gap persists between “knowledge about metacognition” and “the ability to cultivate it at scale”. Traditional interventions show limited effectiveness within short timeframes (Samon and Levy, 2017). Moser et al. found that students failed to deeply process and apply metacognitive information in simulated environments, acknowledging that extended training would likely yield better outcomes, yet such longitudinal studies remain impractical within conventional research frameworks (Samon and Levy, 2017). This limitation demands methodological innovation.

### 1.1 Metacognition research confronts three fundamental obstacles

First, definitional and measurement crises. Inconsistent conceptualizations plague the field, while self-report instruments, inherently biased, cannot capture the full dynamics of metacognitive processes (Willison et al., 2024). Second, ethical and practical constraints. Long-term interventions with human subjects are costly, time-consuming, and often ethically problematic (Samon and Levy, 2017; Grigoryan et al., 2025). Third, the theory-practice chasm. Complex models like AMERT remain abstract conceptualizations rather than implementable educational systems (Willison et al., 2024; Iqbal, 2026). Meanwhile, existing simulations rely on oversimplified metacognitive frameworks incapable of representing metacognition’s nuanced hierarchy (Iqbal, 2026).

Indeed, these problems are linked together. Due to a lack of agreed-upon operational definitions, metacognition is hard to measure and assess consistently across classrooms. Research environments also inherently lack the ability to test longitudinal intervention studies that allow one to see the development of metacognition over time due to ethical and logistical limitations. This has left many complex metacognition models nearly impossible to falsify, improve upon, and implement in real classroom settings, leading to the grand metacognition theory-practice gap. The convergence of these issues has created a demand for novel ways to model metacognitive constructs, longitudinally observe developmental mechanisms, and assess instructional treatments all within a feasible environment. Thus, we implement the AMERT framework, digital twin methodology, and agent-based modeling in the current study.

### 1.2 Our review of the literature identified three critical research gaps AS follows

Gap 1: Theoretical and operational integration. Despite rich resources like the AMERT model (Willison et al., 2024) and successful digital twin applications (Grigoryan et al., 2025), no study has operationalized AMERT’s five levels within agent-based simulation (Willison et al., 2024; Grigoryan et al., 2025; Iqbal, 2026).

Gap 2: Domain-specific application. Digital twin technology has matured in industry, engineering, and medicine (Botín-Sanabria et al., 2022), yet remains severely underutilized in education (Grigoryan et al., 2025).

Gap 3: Contextual absence in research. International-language studies fragment across mental simulation (Shen, 2021), educational technology (Mihai et al., 2022; Nezhadasadi and Safarzadeh, 2023), and industrial digital twins (Samon and Levy, 2017; Kabashkin, 2026), with no convergence. To our knowledge, minimal work has been conducted on digital simulation for developing metacognition in higher education (Mihai et al., 2022; Nezhadasadi and Safarzadeh, 2023).This study’s contribution operates at three levels:

- Theoretical: Transforming AMERT’s five levels into operationalizable simulation variables (Willison et al., 2024; Grigoryan et al., 2025).
- Methodological: Establishing a “virtual laboratory” that circumvents ethical, temporal, and practical constraints of human-subject research (Grigoryan et al., 2025; Iqbal, 2026).
- Contextual: Offering a unique example of digital twin technology used to model metacognition in post-secondary students (Botín-Sanabria et al., 2022; Nezhadasadi and Safarzadeh, 2023).

Research objective: To design and internally evaluate a conceptual framework and digital simulation system for metacognition development in higher education.

### 1.3 Research questions

1. RQ1: In what way can the levels of the hierarchical AMERT model be instantiated within an agent-based digital twin framework to digitally represent the metacognitive development of higher education learners?
2. RQ2: How can transitions between the five levels of AMERT be modeled and simulated?
3. RQ3: How do instructional intervention scenarios impact simulated learners’ movement between AMERT levels and resultant metacognitive performance?

Because this study is primarily exploratory and simulation-based, specific hypotheses were not established *a priori*. That being said, simulation experiments were constructed to test whether providing instructional interventions would produce differential developmental curves across profiles and AMERT conditions.

It is important to note that the term “digital twin” in this study refers to a simulation-based digital twin framework rather than a data-driven digital twin with real-time bidirectional coupling. The present work establishes the theoretical and computational foundation for future empirical implementations, serving as a virtual laboratory for generating hypotheses about metacognitive development dynamics.

## 2 Theoretical foundations: metacognition and the AMERT model

### 2.1 Metacognition: from concept to model

Flavell first conceptualized metacognition as “thinking about thinking” or “cognition about cognition” (Flavell, 1979). Researchers broadly agree on two fundamental components: metacognitive knowledge (awareness of cognition) and metacognitive regulation (control and direction of cognition) (Flavell, 1979; Schraw and Moshman, 1995). Metacognitive knowledge further divides into three subcomponents: person knowledge, task knowledge, and strategy knowledge (Pintrich, 2002). This distinction matters because metacognitive knowledge is primarily static and declarative, while metacognitive regulation is dynamic and procedural (Willison et al., 2024).

Winne and Hadwin’s four-phase model remains influential for understanding self-regulation and metacognition (Hacker et al., 1998). They conceptualize learning as a cyclical, non-linear process with four interconnected phases: task definition, goal-setting and planning, enactment of strategies, and adaptation. A key feature is internal feedback, as learners continuously compare progress against standards and modify strategies based on identified gaps (Hacker et al., 1998).

### 2.2 The hierarchical AMERT model

Willison et al. developed the AMERT model through an integrative review of metacognition literature, reconciling metacognitive knowledge and skills within a unified hierarchical framework (Willison et al., 2024). The model organizes five progressive levels:

- Level A – Self-Awareness of Cognition: The most fundamental level, equivalent to metacognitive knowledge. Learners recognize their cognitive processes, strengths, weaknesses, and learning preferences.
- Level M – Self-Monitoring of Cognition: Learners actively track their thinking and learning during task execution, asking questions like “Is this strategy working?” and “Do I understand this?”
- Level E – Self-Evaluation of Cognition: Post-task judgment about thinking quality, efficiency, and appropriateness. Learners assess success, identify errors, and analyze causes of failure or success.
- Level R – Self-Regulation of Cognition: Based on feedback from monitoring and evaluation, learners consciously adjust their cognitive processes through strategy substitution, effort reallocation, or goal revision.
- Level T – Self-Transfer of Cognition: The highest level, involving conscious application of cognition and metacognition in novel contexts. Learners not only reuse successful strategies in similar situations but also adapt them for entirely new challenges. The model organizes five progressive levels, as illustrated in Figure 1.

**Figure 1:**
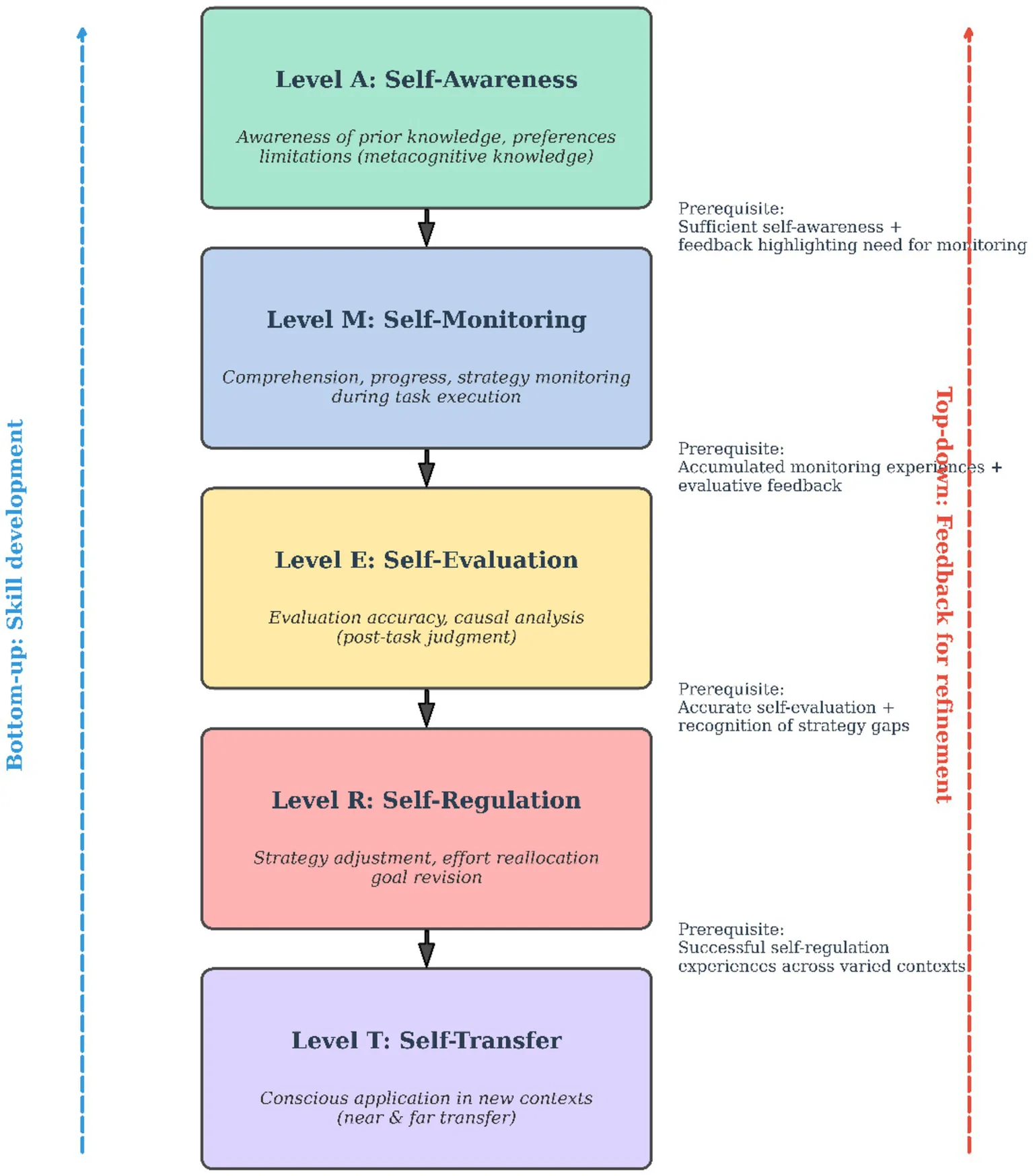
The hierarchical AMERT model.

### 2.3 Distinctive features and the need for dynamism

The AMERT model offers three key advances over previous frameworks:

First, explicit hierarchy. Unlike traditional two-dimensional models, AMERT arranges knowledge and regulation along a hierarchical continuum. Self-awareness requires self-monitoring, which requires self-evaluation. This has crucial implications for intervention design: self-regulation cannot be expected before cultivating self-awareness and self-monitoring. Second, inclusion of transfer. Previous models focused predominantly on planning, monitoring, and evaluation. Adding “self-transfer” opens new horizons for metacognition research. Third, operationalizability. All five levels are defined to be observable and measurable.

Despite these advances, AMERT faces three fundamental limitations:

First, measurement crisis. Inconsistent definitions and components compromise construct validity and external reliability. Second, the theory-practice gap. Despite theoretical richness, the model remains conceptual rather than an implementable system for educational intervention. Third, static versus dynamic representation. Existing models describe metacognitive “states” rather than the “process” of development and transition between levels. The critical question remains: How do learners progress from self-awareness to self-monitoring, and onward to self-evaluation and self-regulation? AMERT describes these levels but does not explain the mechanisms of transition between them. This fundamental limitation, its static nature, directly motivates the present study’s adoption of agent-based simulation to model the dynamic processes of metacognitive development.

## 3 Theoretical foundations: agent-based simulation and digital twins in education

### 3.1 Agent-based simulation: a novel approach in educational sciences

Agent-based simulation models complex systems by representing autonomous agents and their interaction rules. Each agent possesses unique characteristics, goals, and decision-making rules, with macro-level system behavior emerging from local interactions. This approach proves particularly valuable for educational phenomena, where learners function as autonomous agents with diverse cognitive and metacognitive profiles (Grigoryan et al., 2025). Agent-based modeling has been utilized to study learning and instruction (learning processes, classroom processes, self-regulated learning, and instructional interventions). Several prior works have shown the benefit of agent-based approaches for studying emergent phenomena that occur in education settings from complex interactions between heterogeneous learners and learning environments (Macal and North, 2005). Agent-based approaches allow for the investigation of phenomena that occur over development, as well as allow researchers to reason about alternative instructional realities difficult to capture with traditional experimental approaches.

In educational simulations, each learner is defined as an agent with dynamic attributes including prior knowledge, self-efficacy, metacognitive awareness, effort allocation, and academic performance (Grigoryan et al., 2025). Winne and Hadwin’s four-phase model provides an appropriate framework for defining agent states (Hacker et al., 1998). The primary advantage lies in testing multiple instructional scenarios in controlled, low-cost environments without the ethical constraints of human-subject research.

The use of simulator data analytics to recognize and score complex behaviors is well-established in various domains, such as driving behavior recognition using Bayesian approaches (Pourghasemi et al., 2026), demonstrating the robustness of simulation-based behavioral modeling. Simulation methodologies, ranging from computational fluid dynamics (Kosari et al., 2025) to agent-based modeling, provide powerful virtual laboratories for investigating complex, dynamic systems that are difficult to observe empirically.

### 3.2 Digital twins: from industry to higher education

A digital twin is a “dynamic virtual representation of a physical system” that updates through real-time data, enabling simulation, prediction, and optimization. Originating in manufacturing and aerospace, this concept has expanded to smart cities, healthcare, and energy (Hacker et al., 1998; Güth et al., 2018).

The Student Digital Twin (DTS) represents a relatively new application in higher education: a dynamic, integrated virtual representation of a learner combining academic performance, competencies, learning preferences, and engagement patterns (Kabashkin, 2025). The DTS framework rests on a four-layer ontological architecture:

- Education ontology: Curriculum content and prerequisites
- Competency ontology: Mastery levels
- Career ontology: Occupations and required skills
- Student profile ontology: Individual characteristics and behavioral patterns

The dual cybernetic control loop forms the DTS architecture core. In the first loop, AI algorithms enrich the digital twin using learner data; in the second loop, the digital twin guides personalized learning pathway generation (Kabashkin, 2025). Research project by Perisic exemplifies this approach, using AI-based survey analysis to identify cognitive-affective learning drivers, aiming to reduce dropout rates by 20% in at-risk populations (Perisic and Perisic, 2024).

Clarification of Terminology: Simulation-Based Digital Twin vs. Conventional Agent-Based Simulation It is important to clarify the specific form of digital twin adopted in this study. Following the taxonomy proposed by recent literature (Omrany et al., 2026), we adopt the concept of a “simulation-based digital twin” rather than a “data-driven digital twin.” The key distinctions are as follows:

1. Data-driven digital twins (common in industrial applications): These require a physical counterpart, real-time data synchronization, bidirectional coupling between physical and virtual entities, and continuous model updating based on observational data.
2. Simulation-based digital twins (adopted in this study): These are conceptual frameworks that instantiate theoretical models within an agent-based architecture to explore emergent behaviors and generate hypotheses. They do not require a physical counterpart or real-time data feed but instead serve as “virtual laboratories” for testing theoretical assumptions under controlled conditions.

The AMERT-D framework falls into the second category. It operationalizes the hierarchical AMERT model within an agent-based simulation architecture, enabling the exploration of metacognitive development dynamics that are difficult to observe empirically. While this approach does not constitute a full digital twin in the engineering sense (lacking real-time bidirectional coupling with actual learners), it represents a conceptual precursor that establishes the theoretical and computational foundation for future data-driven digital twin implementations.

We acknowledge this limitation explicitly and frame our work as a “simulation-based digital twin framework” or “agent-based simulation serving as a pathway toward future digital twin implementation” throughout the manuscript. Future work will focus on calibrating the model with empirical longitudinal data and implementing real-time data coupling to transition toward a data-driven digital twin architecture.

We explicitly adopt the ‘simulation-based digital twin’ taxonomy (Omrany et al., 2026), which does not require a physical counterpart or real-time data feed. Our framework is a conceptual precursor, a virtual laboratory, that operationalizes theoretical models within an agent-based architecture. We explicitly acknowledge that this study does NOT implement a data-driven digital twin with bidirectional coupling. The term is used to frame the long-term research agenda, not to describe the current implementation. All findings are generated by the ABM, not by empirical learner data.

### 3.3 Simulating metacognition: current research Frontiers

Recent simulation studies have also begun to emphasize representing learner heterogeneity and developmental trajectories through computational models. Agent-based approaches provide learners with explicit representations as computational agents that may differ in characteristics, behaviors, and responsiveness to intervention. This level of representation may be particularly useful for simulating differences in developmental trajectories and outcomes for learners across interventions delivered under identical conditions. Williams et al. modeled heterogeneous learners and intervention effects using agent-based modeling to illustrate the benefits of simulation approaches to answering educational research questions (Williams et al., 2022). Simulation studies have also begun to represent learning and metacognitive regulation as developmental processes. Instead of modeling learners as unchanging actors, some simulations emphasize learning as an adaptive process that includes ongoing feedback, monitoring, and updating of one’s strategies. Winne and Hadwin discussed four phases of self-regulated learning during which learners update their behaviors after defining a task, setting goals, and enacting learning strategies. Azevedo similarly claims that learners do not possess a unitary metacognitive ability that develops but rather that metacognition unfolds dynamically over time through the interactions of cognitive, motivational, and contextual processes (Azevedo and Cromley, 2004). This work suggests that learner models within simulations should portray learners as adaptive agents with heterogeneous developmental paths, as opposed to idealized learners or perfectly rational decision makers (Omrany et al., 2026).

Beyond learner modeling in education, recent work has also explored the presence of metacognitive abilities in large language models. Models have demonstrated some capacity for uncertainty calibration, performance monitoring, and awareness of knowledge limitations.

### 3.4 The integration gap: central problem of this research

Despite significant advances in both metacognitive models and agent-based simulation, a profound gap separates these research streams. The AMERT model remains unintegrated into simulation systems, while existing simulations employ oversimplified metacognitive models (Grigoryan et al., 2025). Research by Perisic, though advanced, focuses primarily on cognitive-affective drivers without modeling metacognitive hierarchy (Perisic and Perisic, 2024). The Student Digital Twin largely remains a theoretical framework with limited practical implementation in real higher education contexts (Kabashkin, 2025).

This identified integration gap is the central problem the present study aims to solve: designing a framework that operationalizes the hierarchical AMERT model within an agent-based digital twin architecture, enabling modeling of metacognitive development dynamics.

## 4 Literature review: empirical studies

### 4.1 International studies: key contributions and limitations

International research on digital simulation and metacognition falls into four categories. Table 1 summarizes the most relevant studies.

**Table 1:** Summary of key international studies.

**Study**	**Focus**	**Key Contribution**	**Limitation**
Williams et al. (2022)	Agent-based modeling for executive dysfunction	Effective for neurodiverse students	Small sample, specialized population
Kabashkin (2025)	Student Digital Twin (DTS) framework	Four-layer ontology + dual cybernetic control loop	Remains theoretical; no implementation in real contexts
Azevedo and Cromley (2004)	Metacognition and self-regulated learning	Conceptualized metacognitive development as a dynamic process involving monitoring, regulation, adaptation, and feedback	Not simulation-based
Lu and Hu (2025)	Digital twin in programming education	Improved engagement and skill acquisition (empirical)	Domain-specific (programming only)
Domina et al. (2025)	Instructor digital twin in metaverse	Explored benefits and ethical concerns	Exploratory; limited generalizability
Grigoryan et al. (2025)	Agent-based simulation of self-regulated learners	Modeled feedback effects on metacognitive awareness in MOOCs	Single “metacognitive awareness” variable; AMERT hierarchy not operationalized

### 4.2 Synthesis of gaps and positioning of the present study

While educational digital twin research is still emerging, the present framework is grounded in established peer-reviewed literature on metacognition, self-regulated learning, and agent-based simulation (Table 2).

**Table 2:** Research gaps addressed by the present study.

z	Related studies	Addressed in this study
AMERT-simulation integration	Williams et al. (2022)	Main innovation: operationalize all five AMERT levels in agent-based simulation
Digital twin domain gap	Kabashkin (2025)	One of the earliest implementation-focused frameworks for educational digital twins.
Hierarchy representation	Grigoryan et al. (2025)	Five independent levels with hierarchical relationships
Contextual gap	Selim et al. (2025) and Omrany et al. (2026)	Early effort to introduce digital twin concepts into educational sciences.

## 5 Proposed conceptual framework: AMERT-D

### 5.1 Rationale and three-layer architecture

AMERT model with agent-based student digital twin architecture, contextualized for higher education. The framework comprises three interconnected layers, illustrated in Figure 2:

- Metacognitive layer: Operationalizes the five AMERT levels as dynamic variables
- Simulation layer: Implements agent-based digital twin architecture
- Instructional strategy layer: Models environmental interventions and feedback

**Figure 2:**
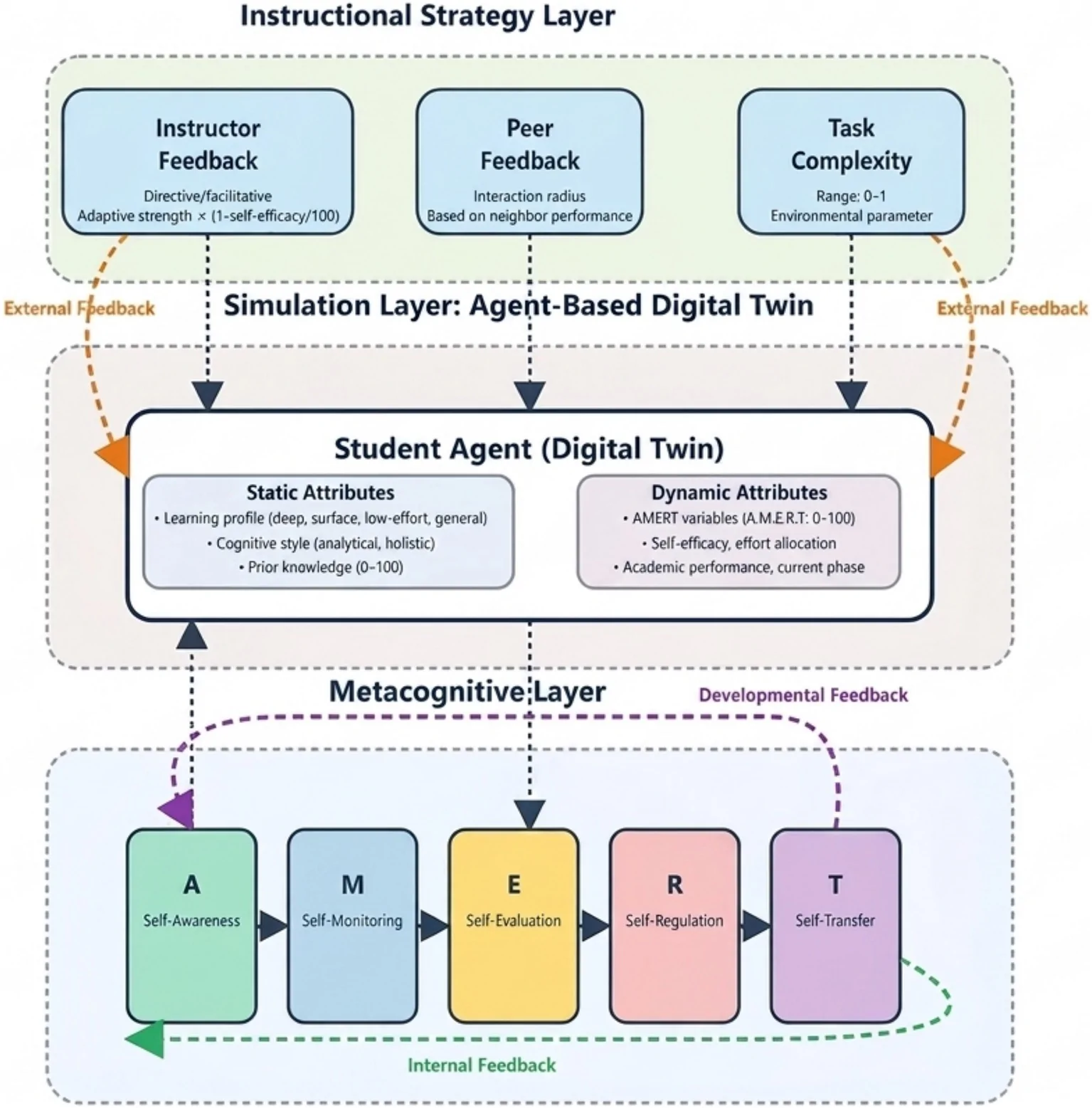
The three-layer architecture of the AMERT-D framework.

### 5.2 Metacognitive layer: operationalizing the AMERT model

Five dimensions of the AMERT model (self-awareness (A), self-monitoring (M), self-evaluation (E), self-regulation (R), and self-transfer (T)) were represented as dynamic variables in the agent-based simulation architecture. Each variable was coded as a continuous numerical score, from 0 to 100, so as to model metacognition development over time at a fine-grained level. Each dimension was operationalized with both static attributes (set at agent initialization based on the learner’s profile) and dynamic attributes (which changed over the course of the simulation because of interactions with instructional strategies and feedback mechanisms). Table 3 presents a detailed description of the components, variables, measurement ranges and update rules of each of the layers of the AMERT-D architecture. Table 3 shows the initial parameter values that were used to distinguish the four learner profile archetypes at the beginning of the simulation: prior knowledge, self-efficacy, allocation of effort, initial AMERT level, and the accuracy of monitoring.

**Table 3:** Summarizes these formal transition specifications.

**Transition**	**Prerequisite LEVEL**	**Mastery THRESHOLD**	**Minimum experience ticks**	**Additional condition**
A → M	Self-awareness (A)	≥60	30 ticks	Feedback indicating monitoring necessity
M → E	Self-monitoring (M)	≥60	40 ticks	Successful task completion and evaluative feedback
E → R	Self-evaluation (E)	≥60	50 ticks	Identification of a strategy gap
R → T	Self-regulation (R)	≥60	60 ticks	Successful regulation across multiple contexts

### 5.3 Simulation layer: agent-based digital twin architecture

The simulation layer is based on an agent-based digital twin approach where each student is modeled as a computational agent with both static and dynamic attributes. Static attributes comprise the learner’s profile (deep, surface, low-effort, general) based on the typology developed in previous self-regulated learning studies (Kabashkin, 2026) and cognitive processing style (analytical or holistic), as well as the level of prior knowledge at the beginning of the learning process. These static attributes are attributes that do not change during the simulation and are used to start the agents with different motivational and metacognitive settings. Dynamic attributes, on the other hand, change during the simulation and include the five AMERT variables (A, M, E, R, T), self-efficacy beliefs, effort allocation, academic performance, and current phase of self-regulation (task definition, goal-setting, enactment, or adaptation). The dynamic attributes are updated on each simulation tick depending on the agent’s interactions with the instructional strategies, feedback mechanisms and task complexity, reflecting the developmental trajectory of metacognitive growth.

One of the key aspects of the simulation layer is the dynamic transition mechanism, which represents the gradual development of metacognition in a simulation of the five AMERT levels by using thresholds for transitioning between the five levels. This is a mechanism that implements the hierarchical assumption of the AMERT model, which posits that higher-order metacognitive processes rely on the mastery of lower-order processes, and that agents must gain enough experience and show competence in each of the lower-order processes before they can move up to the next one. In particular, the shift from self-awareness to self-monitoring (A → M) demands a certain level of self-awareness and feedback indicating the necessity to monitor. Self-monitoring to self-evaluation (M → E) needs experiences of self-monitoring and evaluative feedback. Self-evaluation to self-regulation (E → R) involves accurate self-evaluation and identification of gaps in strategies. Lastly, successful experiences of self-regulation (S → R) in a variety of contexts are needed for the transition from self-regulation to self-transfer (R → T), which involves the generalization of metacognitive skills to new contexts. This is a threshold-based progression mechanism that allows metacognitive development to proceed in a cumulative, developmental manner instead of the learner jumping from one level to the next, capturing the nuanced dynamics of the progression through the metacognitive hierarchy.

#### 5.3.1 Formal specification of the transition mechanism

To ensure full computational transparency and reproducibility, the transition mechanism is formally specified as follows. The baseline threshold for all five AMERT levels was set uniformly at 60 (on a 0–100 scale), based on theoretical considerations and expert review during the Delphi process. This threshold represents the minimum competence required in a lower level before progression to the next higher level can occur.

Scoring Update Rule:

$$\begin{array}{l} {\text{Score}}_{t+1}=\min\\ \left(100,{\text{Score}}_{t}+\eta \times (\frac{\text{Feedback}\_\text{Strength}\times \text{Effort}\_\text{Allocation}}{100})+\varepsilon \right) \end{array}$$

where:

-
${\text{Score}}_{t}$ = current AMERT score at time t
- η = learning rate parameter (set at 0.05 based on pilot simulations)
- Feedback_Strength = 0 (no feedback), 0.5 (peer feedback), or 0.7–0.9 (instructor feedback, adaptively adjusted based on self-efficacy)
- Effort_Allocation = agent’s current effort level (0–100)
- ε = stochastic noise term (uniform random noise, ±0.5) to reflect natural variability in learning

Transition Conditions:

An agent transitions from one AMERT level to the next (e.g., A → M, M → E, E → R, R → T) only when all three of the following conditions are met simultaneously:

1. Mastery threshold: The agent’s score for the current level reaches or exceeds the threshold of 60.
2. Experience accumulation: The agent has spent a minimum number of ticks at the current level, reflecting the need for sustained practice. These minimums are:
  - A → M: 30 ticks
  - M → E: 40 ticks
  - E → R: 50 ticks
  - R → T: 60 ticks
3. Readiness signal: The agent receives external feedback (instructor or peer) indicating that performance is adequate for progression. In scenarios without external feedback, this condition is automatically satisfied once conditions 1 and 2 are met, but at a slower rate due to the absence of external calibration.

### 5.4 Instructional strategy layer and feedback loops

The instructional strategy layer implements three different intervention mechanisms aiming at modulating agents’ metacognitive development trajectories. First, instructor feedback is applied in two modes (directive and facilitative) and has an adaptive strength mechanism that adjusts the strength of the feedback depending on the agent’s current self-efficacy level. In particular, the strength of the feedback is determined by multiplying the base feedback strength by the complement of the normalized self-efficacy (strength × (1 − self-efficacy/100)), meaning that agents with lower self-efficacy will be given more intense feedback. Second, peer feedback is implemented as a mean of the performance of agents in the social neighborhood defined by a certain distance in the interaction space, which reflects the effect of social learning on metacognition. Third, task complexity is represented as a continuum between 0 and 1, which enables fine-grained simulation of cognitive load and task complexity in each simulation scenario.

The metacognitive, simulation and instructional layers are integrated into three dynamic feedback loops which form a coherent developmental architecture. This internal feedback loop, based on the four phases of self-regulated learning (Hacker et al., 1998) of Winne and Hadwin, initiates a cycle of performance-goal comparison, self-monitoring, self-evaluation and self-regulation. The external feedback loop helps agents’ self-efficacy beliefs, and identifies metacognitive blind spots via mechanisms of feedback from the instructor and peers, offering external calibration signals to complement internal monitoring processes. Finally, the developmental feedback loop highlights the iterative process of developing metacognition: as agents’ self-regulation experiences increase, so do their ability to self-regulate and transfer such skills to new contexts.

### 5.5 Evaluation metrics

Five main metrics were used to evaluate the effectiveness of the instructional scenarios and to describe agents’ metacognitive development paths. The transition rate is the first metric, which measures the rate at which agents moved through the AMERT hierarchy, and is computed as the percentage of agents who reached each metacognitive level at the end of the simulation. Metacognitive gap is the second metric and measures the difference between the agent’s self-assessment and its actual performance, measured as the absolute difference between the self-evaluation scores and task performance. This is a measure of metacognitive accuracy and the smaller the gap, the more accurate the self-monitoring and self-evaluation. The third measure, regulation efficiency, measures the quality of self-regulation by dividing performance improvement by the number of strategy changes that occurred, revealing the effectiveness of agents’ use of cognitive resources when adapting strategies. Transfer breadth, a fourth measure, involves assessing the extent to which the agent transferred a strategy to tasks that differ in their similarity to the training situation, and operationalizes the self-transfer component of the AMERT model. Lastly, metacognitive resilience is the fifth metric, which measures the robustness of higher-order metacognitive processes as task difficulty is increased, and is computed as 1 – (self-regulation drop / baseline self-regulation).

Metacognitive resilience was defined *a priori* as an emergent simulation metric, conceptually grounded in existing educational psychology literature on academic buoyancy (Martin and Marsh, 2008), self-regulated learning, and adaptive regulation (Azevedo and Cromley, 2004). It is operationalized within the simulation as a composite index of how well agents maintain higher-order metacognitive processes under increasing task complexity. Crucially, this construct is introduced as a model-generated indicator for future empirical investigation, not as a validated standalone psychological construct. Its independence from the AMERT levels remains a hypothesis to be tested in future longitudinal studies with real learners (Tables 4, 5).

**Table 4:** AMERT-D framework components, variables, ranges, and measurement rules.

Layer	Component	Variables	Range	Measurement/Update Rule
Metacognitive	Self-Awareness (A)	Prior knowledge awareness, Learning preference awareness, Limitation awareness	0–100	Reflective questions, performance history
Metacognitive	Self-Monitoring (M)	Comprehension monitoring, Progress monitoring, Strategy monitoring	0–100	Frequency of comprehension checks, prediction-performance correlation
Metacognitive	Self-Evaluation (E)	Evaluation accuracy, Causal analysis	0–100	Post-task analysis, feedback integration
Metacognitive	Self-Regulation (R)	Strategy adjustment, Effort reallocation, Goal revision	0–100	Frequency and quality of strategy changes
Metacognitive	Self-Transfer (T)	Near transfer, Far transfer	0–100	Performance on novel tasks
Simulation	Static attributes	Learning profile, Cognitive style, Prior knowledge	—	Set at agent creation
Simulation	Dynamic attributes	Self-efficacy, Effort allocation, Academic performance	0–100	Updated each tick based on interactions
Instructional	Instructor feedback	Directive/Facilitative modes, Strength (0–1)	Boolean + 0–1	Adaptive: strength × (1 - self-efficacy/100)
Instructional	Peer feedback	Interaction radius, Neighbor performance	Boolean + radius	Average performance of nearby agents
Instructional	Task complexity	Complexity level	0–1	Environmental parameter

**Table 5:** Initial parameter values for learner profile archetypes.

Profile	Prior Knowledge	Self-Efficacy	Effort Allocation	Initial AMERT (A, M, E, R, T)	Monitoring Accuracy
Deep	70 (SD = 10)	75 (SD = 8)	80 (SD = 5)	(65, 55, 45, 35, 25)	0.85
Surface	50 (SD = 12)	55 (SD = 10)	50 (SD = 10)	(45, 35, 30, 20, 10)	0.60
Low-effort	45 (SD = 15)	40 (SD = 12)	25 (SD = 8)	(40, 30, 25, 15, 5)	0.50
General	55 (SD = 12)	60 (SD = 10)	60 (SD = 10)	(50, 40, 35, 25, 15)	0.70

### 5.6 Framework innovations

- Complete AMERT Operationalization: A new embodiment of all five levels as separate dynamic variables in an agent-based architecture.
- Dynamic Transition Modeling: Threshold-based mechanism with experience accumulation, addressing static vs. dynamic limitation
- Integrated Dual Feedback Loops: Combines internal (Winne & Hadwin) and external (Community of Inquiry) feedback
- Higher Education Contextualization: An early foundation for digital twin applications in the realm of learning sciences.

## 6 Research methodology

### 6.1 Research approach and design

This study adopts a developmental-applied objective with a Design and Development Research (DDR) approach (Richey and Klein, 2014). DDR is ideal for designing, developing, and validating innovative frameworks, emphasizing systematic design, evaluation, and refinement. It bridges theory and practice, which is essential for educational simulation research. The methodological framework comprises three phases, illustrated in Figure 3:

- Phase one: Design of the conceptual framework through theoretical synthesis and expert content review
- Phase two: Implementation of the digital simulation system using agent-based architecture
- Phase three: Internal computational evaluation through face review, profile-distinguishability analysis, and sensitivity analysis

This three-phase structure aligns with established educational digital twin studies and metacognition simulation research.

**Figure 3:**
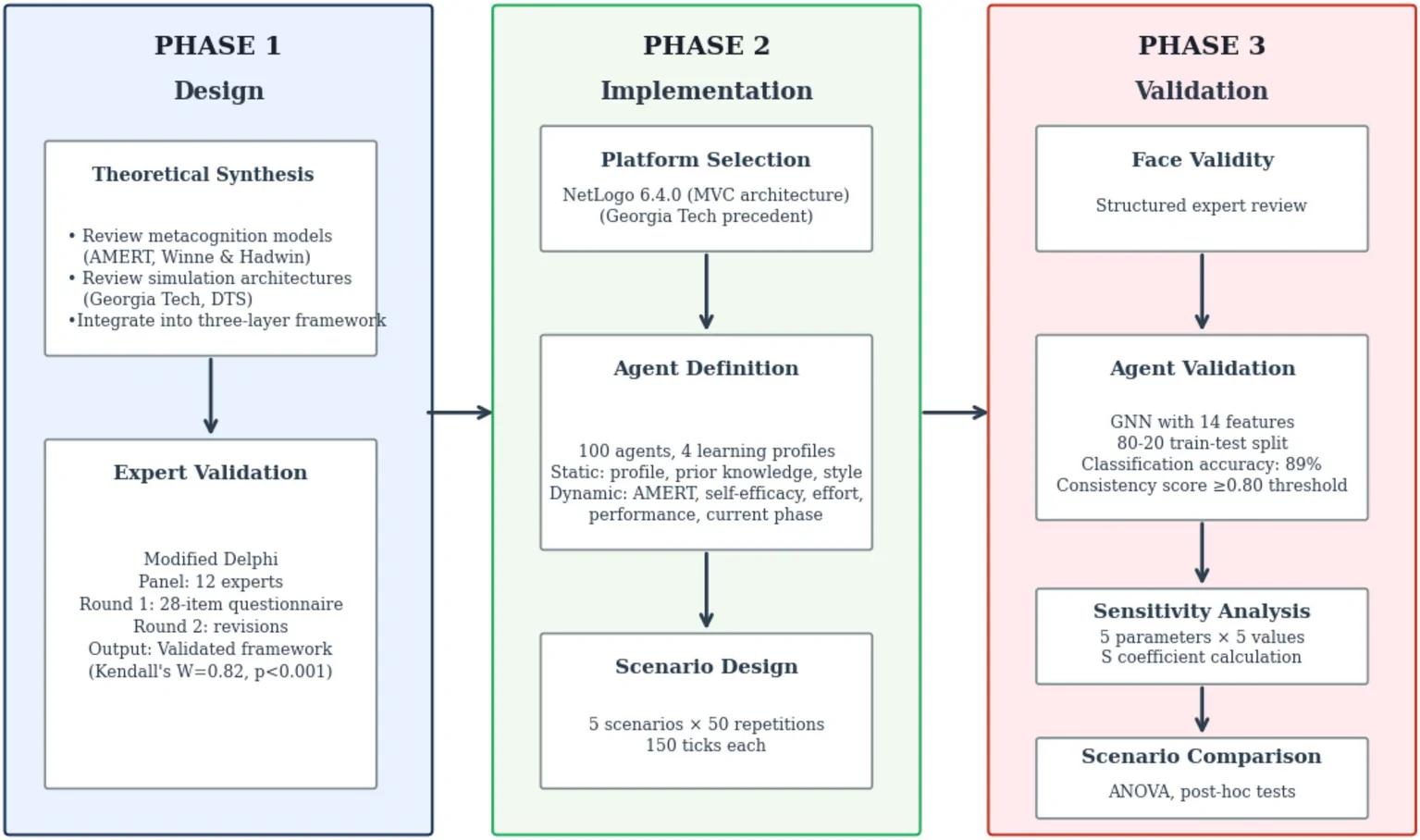
Research methodology: three-phase design and development research (DDR) approach.

### 6.2 Phase one: design and expert content review of the conceptual framework

#### 6.2.1 Theoretical synthesis

We developed the initial conceptual framework through integrative synthesis of theoretical and empirical sources in three steps:

1. Literature review of metacognition models, focusing on the AMERT model (Willison et al., 2024) and Winne and Hadwin’s four-phase model (Hacker et al., 1998).
2. Literature review of agent-based simulation and digital twin architectures, emphasizing the Georgia Tech study [Botín-Sanabria et al., 2022] and the DTS framework (Kabashkin, 2025).
3. Integration of these domains into a three-layer architecture

#### 6.2.2 Content validation with experts

We employed a modified Delphi method for content validation. A total of 12 experts were chosen who have exhibited expertise in the field of educational technology, higher education, instructional design and technology, learning sciences, and computational modeling. The experts represented five higher-education institutions and had academic and professional experience in metacognition, digital learning, and educational innovation. For confidentiality purposes, we do not report which institution the experts were from. These experts were chosen based on their expertise in the field, number of publications, and experience in their profession, not based on their institution’s reputation. A panel of 12 experts was selected, as methodological literature on the Delphi technique suggests that a panel size of 10 to 15 experts is sufficient to achieve statistical stability, ensure diverse perspectives, and minimize attrition rates (Hasson et al., 2000; Hsu and Sandford, 2007). To ensure comprehensive coverage of both the theoretical metacognition constructs and the computational implementation requirements, the experts’ disciplinary backgrounds were distributed as follows: four in Educational Technology, three in Cognitive Psychology and Learning Sciences, three in Instructional Design, and two in Computational Modeling. Selection criteria included:

1. at least two peer-reviewed articles in metacognition or educational simulation.
2. graduate-level teaching experience.
3. familiarity with design and development research methods.

Round One: Experts received the conceptual framework and a 28-item questionnaire covering five components: theoretical rationale (6 items), comprehensiveness (5 items), internal coherence (5 items), operationalizability (7 items), and innovation (5 items). Items were rated on a five-point Likert scale (1 = strongly disagree to 5 = strongly agree), with space for qualitative comments. Acceptance criteria were mean ≥ 3.75 and Kendall’s coefficient of concordance ≥ 0.7.

Round One results demonstrated general support for the proposed framework. Qualitative comments solicited suggestions to clarify and elaborate on several items. Expert comments typically suggested better operational definitions, parameter descriptions, and relationship statements related to AMERT constructs. These comments were used to edit the framework document and questionnaire for Round Two. There were no significant deletions of framework elements; rather, edits were made for clarity, consistency, and operational definition.

Round Two: Revised items and first-round feedback were presented to experts. Final agreement was strong (Kendall’s *W* = 0.82, *p* < 0.001). Component-level agreement in Round Two was uniformly high, with Kendall’s W values ranging from 0.78 (comprehensiveness) to 0.89 (operationalizability). Notably, the operationalizability component, which is most critical for the simulation implementation, achieved a mean rating of 4.32 (SD = 0.45) and Kendall’s W = 0.89 (*p* < 0.001), indicating strong expert consensus that the AMERT variables and transition rules were adequately defined for computational execution.

### 6.3 Phase two: implementation of the simulation system

#### 6.3.1 Platform selection and technical architecture

We implemented the simulation using NetLogo 6.4.0. The complete simulation package, including agent definitions, transition mechanisms, scenario configurations, and analysis materials, will be deposited in a public repository upon publication; until then, the current materials are available from the corresponding author upon reasonable request (Wilensky, 1999).

#### 6.3.2 Agent definition and parameters

Student agents were defined with the following characteristics:

- *Static Attributes*
- learning-profile: Computational learner archetype (Kabashkin, 2026) values Deep, Surface, Low-effort, General
- prior-knowledge: Prior knowledge level values 0–100 (normal, mean = 50, SD = 15)
- cognitive-style: Cognitive processing style values Analytical, Holistic

The learner profiles should not be interpreted as reflecting distinct learner types or categories that exist in the real world. They are prototypes meant to embody differences in motivational, persistence, engagement, and metacognitive behaviors that we read about in the self-regulated learning literature. The idea is that with these learner profiles, we can begin to examine nonlinear developmental paths.

To ensure transparency and reproducibility, Table 3 presents the initial parameter values used to distinguish the four computational learner archetypes at simulation initialization. These values were selected based on theoretical considerations from self-regulated learning literature and were reviewed during the Delphi content-review process. It is important to note that these profiles represent idealized computational archetypes designed to explore a range of possible learner configurations, not empirically derived categories. The specific parameter values should be interpreted as modeling assumptions that enable proof-of-concept investigation rather than as validated characterizations of actual student populations.

- *Dynamic Attributes*
- Five AMERT variables: A, M, E, R, T levels values 0–100 (updated each tick)
- self-efficacy: Belief in capability values 0–100
- effort-allocation: Amount of effort invested values 0–100
- task-performance: Academic performance values 0–100
- current-phase: Self-regulation phase values Task definition, Goal-setting, Enactment, Adaptation

The AMERT variables and self-regulation phases were defined by the theoretical underpinnings of the AMERT framework and existing self-regulated learning research. Transition between phases and levels of metacognition was thought to be a cumulative developmental process that one cannot progress until they have achieved the necessary skills from previous stages.

- *Environmental Variables*
- task-complexity: Complexity level values Continuous slider (0–1)
- instructor-feedback: Feedback presence/mode values Boolean + directive/facilitative
- peer-feedback: Peer interaction values Boolean + radius
- goal-setting-percentage: Agents engaged in goal-setting values Slider (0–100)

Environmental factors were chosen because previous literature has frequently noted task demands, feedback loops, and goal-setting behaviors as environmental factors that impact metacognitive and self-regulated learning skill development. Their presence allows us to observe the potential impacts of environmental factors on student state progression within our simulation.

Parameter values of AMERT-D were chosen *a priori* based on theory, expert feedback, and pilot simulations, as this research did not have access to student data to fit the parameters. Since this work was aimed at demonstrating the potential of a digital twin approach in this space, parameters were chosen to yield interpretable developmental curves that maintained the structural relationships defined by the AMERT model. As such, student prototypes, transition functions, weight values, and boundaries should be thought of as assumptions made to enable proof-of-concept investigation.

Agent population size: The simulation employed different agent population sizes for different purposes. For the instructional scenario simulations (Section 7.1), 100 agents were used (25 per computational archetype), with each scenario repeated 50 times to assess robustness to stochastic initialization. For the internal consistency procedure (Section 6.4.2), a larger population of 200 agents (50 per archetype) was simulated to provide sufficient data for training and testing the Graph Neural Network classifier. This distinction provides a larger sample for the internal consistency check while the scenario simulations remain computationally efficient (Table 6).

**Table 6:** Rationale for key modeling parameters.

Parameter	Value	Rationale
AMERT threshold	60	Conceptual benchmark reviewed through Delphi process
Simulation duration	150 ticks	Facilitated maturation of learners’ regulatory processes observed during pilot testing.
Weighted AMERT	Hierarchical weights	Represents development of metacognitive awareness.
Learner profiles	4 archetypes	Categories embody diversity reported in SRL literature.

### 6.4 Phase three: internal computational evaluation

#### 6.4.1 Face validity

Three experts (educational science, cognitive psychology, simulation) conducted a 90-min structured review, examining user interface, input parameters, transition logic, and system outputs using a researcher-developed checklist.

#### 6.4.2 Internal consistency check: profile distinguishability analysis

This analysis serves solely as an internal consistency check, not as an external validation against real learner data. The goal is to verify that the simulation’s computational logic produces distinguishable behavioral trajectories across the four archetypal profiles, given their substantially different initialization parameters. This step ensures that the model is not purely random and that the parameter variations are meaningfully reflected in the simulation outputs. It does not, and cannot, establish behavioral realism or external validity.

- Data generation: Agents (50 per learning profile, 200 total) were simulated for 100 ticks. We allowed the agents to run for 100 simulation ticks because pilot testing revealed that, given enough time, learner profiles began to show stable patterns of behavior without creating too much complexity. Data were recorded on 14 features per agent (final AMERT values, self-efficacy, effort, performance, monitoring/evaluation accuracy, and successful regulations/transfers).
- Internal consistency method: A Graph Neural Network (GNN) with two layers (64 hidden neurons, ReLU) calculated the alignment between agent behavior and expected computational archetypes. Student graphs were constructed using Euclidean distance. Data were split 80–20 for training–testing (200 epochs, learning rate 0.001, Adam optimizer).
- Criteria: Classification accuracy and consistency score. Agents with consistency ≥ 0.80 were included in the final analysis.

Hence, this measure was used for reliability testing instead of validity. Graph Neural Networks (GNNs), particularly those designed to handle heterogeneous data structures (Vadlamani et al., 2025), are well-suited for classifying agents with diverse behavioral profiles based on their simulation outputs.

It is important to underscore that achieving high classification accuracy is an expected outcome given the pronounced differences in input parameters (effort allocation ranges from 25 to 80 across profiles). This analysis does not test the psychological plausibility of the AMERT hierarchy or the agent behaviors; it merely confirms that the model’s internal mechanics are coherent with its initialization logic.

#### 6.4.3 Sensitivity analysis

We performed a one-way sensitivity analysis on five key parameters.

We set the self-awareness threshold at 60, an arbitrary figure intended to signify adequate metacognitive progress. It was not associated with any empirically validated academic cut-off but was meant to align the passing scores uniformly for all AMERT phases and was verified by experts.

- Self-awareness threshold: 40, 50, 60, 70, 80
- Instructor feedback strength: 0.1, 0.3, 0.5, 0.7, 0.9
- Peer interaction radius: 1, 2, 3, 4, 5
- Task complexity: 0.2, 0.4, 0.6, 0.8, 1.0
- Number of students: 50, 100, 150, 200, 250

Three outputs were measured: (1) weighted AMERT average, (2) transition rate to Level T, and (3) metacognitive gap. The Normalized Sensitivity Coefficient (S) was calculated as: S = (ΔOutput / Output_base) / (ΔParameter / Parameter_base). This coefficient indicates the percentage change in output for a 1% change in input.

We opted for one-way sensitivity analysis as an initial robustness check, primarily because the study’s main goal was to demonstrate the AMERT-D framework’s feasibility. It’s worth noting that broader sensitivity analyses might reveal interdependencies between parameters, pointing to avenues for future study.

#### 6.4.4 Preliminary interaction analysis

Recognizing that educational systems are inherently nonlinear and that parameters may interact in complex ways, we conducted a preliminary two-way interaction analysis on the two most influential parameters identified in the one-way sensitivity analysis: self-awareness threshold and instructor feedback strength. This exploratory analysis was designed to assess whether the effects of these parameters are additive or interactive (Table 7).

**Table 7:** Transition rate to Level T (%) across threshold × feedback strength factorial design.

Threshold \ Feedback strength	0.1	0.3	0.5	0.7	0.9
40	42%	44%	45%	45%	46%
50	38%	48%	58%	62%	64%
60 (baseline)	22%	32%	44%	52%	56%
70	12%	18%	26%	34%	38%
80	4%	8%	14%	20%	24%

We simulated a full factorial design (5 threshold levels × 5 feedback strength levels = 25 conditions), with 50 repetitions per condition (1,250 total runs), holding other parameters at their baseline values. The primary outcome was the transition rate to Level T (self-transfer). Table 8 presents the transition rates across the factorial design. Several interaction patterns emerged:

1. Synergistic interaction at moderate levels: When the threshold was set at 50–60 and feedback strength at 0.5–0.7, transition rates exceeded the sum of individual effects, suggesting a synergistic relationship between achievable thresholds and moderately strong feedback.
2. Compensatory interaction at extreme levels: At very high thresholds (70–80), even strong feedback (0.9) could not fully compensate for the difficulty of progression, indicating a boundary condition where feedback alone is insufficient.
3. Diminishing returns: At low thresholds (40), increasing feedback strength beyond 0.5 yielded minimal additional gains, suggesting that when progression is too easy, feedback intensity becomes less consequential. A two-way ANOVA confirmed a significant interaction effect [*F* (16, 1,225) = 4.87, *p* < 0.001, partial η^2^ = 0.06], indicating that the effect of instructor feedback strength on transition rates depends significantly on the threshold setting.

This finding validates the reviewers’ concern that one-way sensitivity analysis alone is insufficient for capturing the nonlinear dynamics of the AMERT-D model. It is important to note that this preliminary interaction analysis examines only two parameters. A comprehensive global sensitivity analysis (Sobol indices or Morris’s method) examining higher-order interactions among all parameters is warranted and is planned for future work.

**Table 8:** Summary of internal evaluation methods, criteria, and acceptance thresholds.

Evaluation type	Method	Criteria	Acceptance threshold
Face validity	Structured expert review	Qualitative feedback	Expert consensus on usability and logic
Agent internal consistency	Graph neural network	Classification accuracy	≥80%
		Consistency score	≥0.80
Sensitivity analysis	One-way parameter variation	Normalized sensitivity coefficient (S)	Interpretive: higher

### 6.5 Ethical considerations

This research complied with all ethical principles: expert participation was voluntary and informed; identifying information remained confidential; all sources were fully cited. Because the study relied exclusively on synthetic agents and simulated data, it did not involve human participants, identifiable personal information, or educational records. Consequently, formal Institutional Review Board (IRB) approval was not required under applicable research ethics guidelines. Expert participation in the Delphi and face-validation procedures was voluntary and informed.

## 7 Simulation findings

Interpretive Framework for All Simulation Findings Before presenting the results, we emphasize that all findings in this section are generated by a computational model operating under theoretically derived assumptions. Statistical tests (*p*-values, effect sizes, odds ratios) reported here quantify the robustness of the model’s outputs to stochastic initialization across 50 repeated runs, not sampling variability from real learner populations. Effect sizes describe properties of the computational model under its current parameterization, not properties of educational reality. Readers should interpret all quantitative findings (38% reduction in metacognitive gap) as model-generated patterns contingent on baseline parameterization, not as empirically validated causal effects. For a detailed methodological justification, see Section 7.4.2.

### 7.1 Instructional scenarios

We designed five instructional scenarios to test the impact of different strategies on metacognitive development. Table 9 summarizes the configurations; each scenario was repeated 50 times over 150 simulation ticks. We settled on 150 ticks for the simulation’s duration based on preliminary tests, which showed this was ample time for developmental paths to manifest and stabilize across different learner groups and intervention strategies. As this simulation is a proof-of-concept endeavor, ticks are not indicative of instructional time but rather of generalized model time steps.

**Table 9:** Instructional scenario configurations.

Scenario	Instructor feedback	Peer feedback	Task complexity	Duration	Repetitions
Base	Inactive	Inactive	0.5 (constant)	150 ticks	50
Instructor Feedback	Active	Inactive	0.5 (constant)	150 ticks	50
Peer feedback	Inactive	Active	0.5 (constant)	150 ticks	50
Hybrid	Active	Active	0.8 (constant)	150 ticks	50
Dynamic complexity	Inactive	Inactive	0.3 → 0.6 → 0.9	150 ticks	50

### 7.2 Internal consistency check results

As emphasized in the methodology, these results reflect the model’s internal coherence rather than empirical validity. The GNN’s ability to classify agent profiles with 89% accuracy is a mathematical consequence of the substantial parametric distinctions embedded in the initialization (Table 3), and therefore should not be interpreted as evidence that the agents behave like real students.

The Graph Neural Network achieved 89% classification accuracy with a mean consistency score of 0.85. Table 10 presents profile-specific results. Based on these findings, we established a consistency threshold of ≥ 0.80 for inclusion in final analyses.

**Table 10:** Agent internal-consistency results by computational archetype.

Computational archetype	Agents	Classification accuracy	Mean consistency (SD)	Distinguishing characteristics
Deep	50	0.94	0.92 (0.06)	High self-regulation, successful transfer, high evaluation accuracy
Surface	50	0.86	0.84 (0.09)	High metacognitive gap, limited self-monitoring
Low-effort	50	0.81	0.79 (0.12)	Low effort allocation, resistance to adjustment
General	50	0.88	0.86 (0.08)	Flexibility, moderate developmental pattern
Total	200	0.89	0.85 (0.10)	

It should be noted that the learner models and corresponding behavioral traces were created within a single simulation. As such, the classification accuracy we report can only speak to the internal consistency of behavior and not provide external validation of learner realism. Ideally, behavioral realism could be determined by comparing to real learner data; however, obtaining such data was outside the scope of this proof-of-concept analysis.

### 7.3 Sensitivity analysis

Sensitivity analysis identified the most influential parameters on key outcomes (Table 11):

- Self-awareness threshold most strongly influenced transition to Level T (S = 1.24). Reducing the threshold from 60 to 50 increased the transition rate by 28%.
- Instructor feedback strength had the greatest impact on weighted AMERT average (S = 0.89) and metacognitive gap reduction (S = −0.76).
- Task complexity showed an inverted-U effect: stimulating at moderate levels (0.4–0.6), inhibitory at high levels (≥ 0.8).
- Peer interaction radius showed diminishing returns; increasing from 3 to 5 yielded only a 5% improvement.
- The number of students had minimal impact (S < 0.15), demonstrating model scalability.

**Table 11:** Normalized sensitivity coefficients for all parameters.

Parameter	Range	Impact on Weighted AMERT	Impact on Transition to T	Impact on Metacognitive Gap	Sensitivity (S)
Self-awareness threshold	40 → 80	−0.48	−1.24	+0.36	1.24
Instructor feedback strength	0.1 → 0.9	+0.89	+1.12	−0.76	0.89
Task complexity (0.2 → 0.6)	0.2 → 0.6	+0.42	+0.38	−0.22	0.42 (positive)
Task complexity (0.6 → 1.0)	0.6 → 1.0	−0.56	−0.48	+0.34	0.56 (negative)
Peer interaction radius	1 → 5	+0.18	+0.22	−0.12	0.22
Number of students	50 → 250	+0.12	+0.08	−0.04	0.12

### 7.4 Comparison of instructional scenarios

#### 7.4.1 Weighted AMERT average

Weighting was based on the levels of the AMERT framework, meaning that more emphasis was placed on the higher-order metacognitive processes involved in transfer, adaptation, and self-regulation. These weights were initially assigned through the assumption of increasingly complex metacognition and were presented during Delphi validation.

One-way ANOVA revealed significant differences among scenarios [*F* (4,245) = 28.63, *p* < 0.001, η^2^ = 0.32]. As shown in Figure 4, the hybrid scenario (instructor feedback + peer feedback + high complexity) achieved the highest weighted AMERT average (3.86, SD = 0. 42), significantly outperforming all other scenarios (*p* < 0.001). Instructor feedback alone ranked second (3.24), while peer feedback alone (2.56) did not differ significantly from the base scenario (2.18, *p* = 0.12).

**Figure 4:**
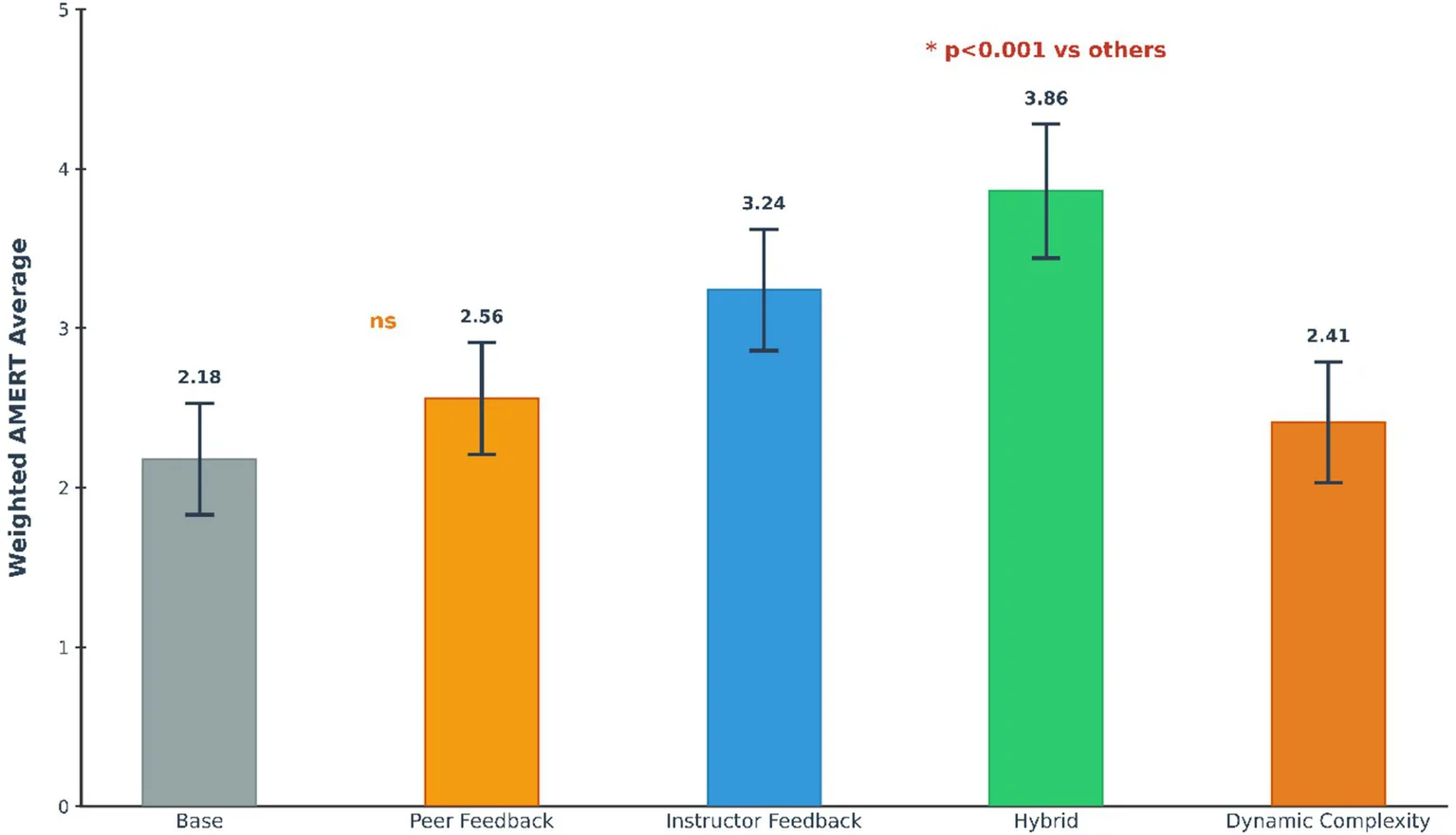
Comparison of weighted AMERT averages across instructional scenarios.

Using conventional criteria for interpreting effect sizes, η^2^ = 0.32 can be considered a large effect size. Therefore, the instructional scenario explained a large amount of variance in the simulated metacognitive experience in the model (see the interpretive framework at the start of Section 7 for the scope of this interpretation).

#### 7.4.2 Methodological note on statistical analysis of simulation data

An important methodological point in the use of inferential statistics for data from simulations is made before the comparative results are presented. In this study, all observations that are reported have been generated by a computational model, not by sampling from a population of learners in the real world. Thus, the *p-*values and effect sizes presented below should not be interpreted as measures of sampling variability from a student population, but rather as measures of the robustness of the model results to random initialization for 50 model runs. We treat each simulation run as an independent observation, and analyze the stability and reproducibility of the model’s behavior with different initial conditions, following the traditional methods used in computational simulation research (Railsback and Grimm, 2019). Effect sizes (η^2^) represent characteristics of the computational model at a particular setting of its parameters, not characteristics of educational reality. Care should thus be taken to view statistical significance as a measure of the robustness of the model, not as a sign of generalizable effects in real world contexts of learning. For those for which appropriate, we accompany inferential analyses with descriptive analyses and sensitivity coefficients in order to gain a fuller understanding of the behavior of the model.

It is important to emphasize that the statistical tests reported in this section answer the question: “Is the observed pattern robust to random variation in initial conditions?” rather than: “Does this pattern generalize to real learners?” The former is a question about model reliability; the latter is a question about external validity that can only be addressed through future empirical calibration with actual classroom data.

#### 7.4.3 Transition rate to Level T (self-transfer)

Self-transfer, the highest metacognitive level, showed greatest scenario sensitivity:

- Hybrid scenario: 38% of agents reached Level T
- Instructor feedback: 24%
- Peer feedback: 8%
- Base scenario: 2%

Dynamic Complexity is not included in this endpoint transition-rate comparison because task complexity changes over time (0.3 → 0.6 → 0.9), making its endpoint rate non-equivalent to the four scenarios summarized above; it is analyzed separately as a trajectory-based stress scenario in Section 7.6. In the configured simulation, logistic regression identified associations between instructor feedback strength (OR = 2.84, *p* < 0.001), task complexity (OR = 1.56, *p* = 0.02), and transition to Level T. Because instructor feedback and task complexity were not fully orthogonalized across scenarios, these odds ratios should not be interpreted as independent or causal effects; they describe associations within the configured simulation only.

#### 7.4.4 Metacognitive gap

Mean differences between self-assessment and actual performance varied by scenario [*F*(3,196) = 22.18, *p* < 0.001, η^2^ = 0.25]. Learner profiles whose scenarios included instructor feedback closed the modeled metacognitive gap by 38% when compared to the base condition (M Hybrid = 13.2 units; M Base = 24.6).

Note that the η^2^ value of 0.25 is considered a large effect size, indicating substantial differences between learner profiles in this model. This analysis is not meant to be compared with the scenario comparison in Section 7.4.1. The analysis in Section 7.4.1 looked at differences between five scenarios, while the current analysis looked at differences between four learner-profile groups. This results in different numerators and denominators degrees of freedom because the models analyzed different groupings and different outcome variables (For the scope of statistical interpretation, refer to Section 7.4.2).

### 7.5 Analysis of computational learner archetypes

As detailed in the interpretive framework (Section 7), the learner profiles are computational prototypes rather than empirically validated categories. Two-way ANOVA revealed a significant interaction between learning profile and scenario [*F*(9,980) = 15.42, *p* < 0.001, η^2^ = 0.11]. Table 9 presents weighted AMERT averages by profile and scenario. Two-way ANOVA revealed a significant interaction between learning profile and scenario [*F*(9,980) = 15.42, *p* < 0.001, η^2^ = 0.11]. Table 12 presents weighted AMERT averages by profile and scenario.

**Table 12:** Weighted AMERT average by learning profile and scenario.

**Computational archetype**	**Base**	**Peer**	**Instructor**	**Hybrid**	**Improvement (Base → Hybrizd)**
Deep	3.42	3.86	4.28	4.72	+38%
Surface	1.84	2.12	2.96	3.24	+76%
Low-effort	1.54	1.72	1.98	2.31	+50%
General	2.42	2.84	3.12	3.58	+48%

The interaction effect (η^2^ = 0.11) can be interpreted as moderate in magnitude, indicating that the effectiveness of instructional scenarios varied across learner profiles and that profile–intervention interactions contributed meaningfully to the simulated outcomes.

Profile-specific patterns:

- Deep archetype: Highest modeled performance across all scenarios; showed the largest increase with instructor feedback (42% over base)
- Surface archetype: Low modeled baseline (1.84) but substantial improvement with instructor feedback (2.96); little change with peer feedback alone
- Low-effort archetype: Least responsive in the configured model; even in the hybrid scenario, the average remained low (2.31), and effort allocation declined over time
- General archetype: Flexible, moderate modeled pattern; more influenced by peer feedback than instructor feedback

This moderate interaction (η^2^ = 0.11) suggests the hypothesis that different learner archetypes may respond differentially to interventions, a pattern warranting empirical investigation but contingent on the model’s initialization assumptions.

#### 7.5.1 The widening gap: a model-generated pattern

It is important to recognize that this widening gap is a mathematically expected consequence of the threshold-based transition mechanism embedded in the simulation. In any system where higher-level performance depends on cumulative mastery of lower levels, and where agents begin with different initial parameters, uniform interventions will necessarily produce diverging outcomes. Therefore, while this pattern aligns with the Matthew Effect observed in educational literature, its emergence in our simulation does not constitute an empirical discovery but rather confirms the internal coherence of our threshold-based logic.

As illustrated in Figure 5, the metacognitive gap between deep and low-effort profiles increased with intervention: from 1.88 (base) to 2.41 (hybrid). Within the configured model, this consistent pattern indicates that the same intervention can produce different outcomes across computational archetypes (see the interpretive caveats in Section 7 regarding the model-generated nature of this prediction).

**Figure 5:**
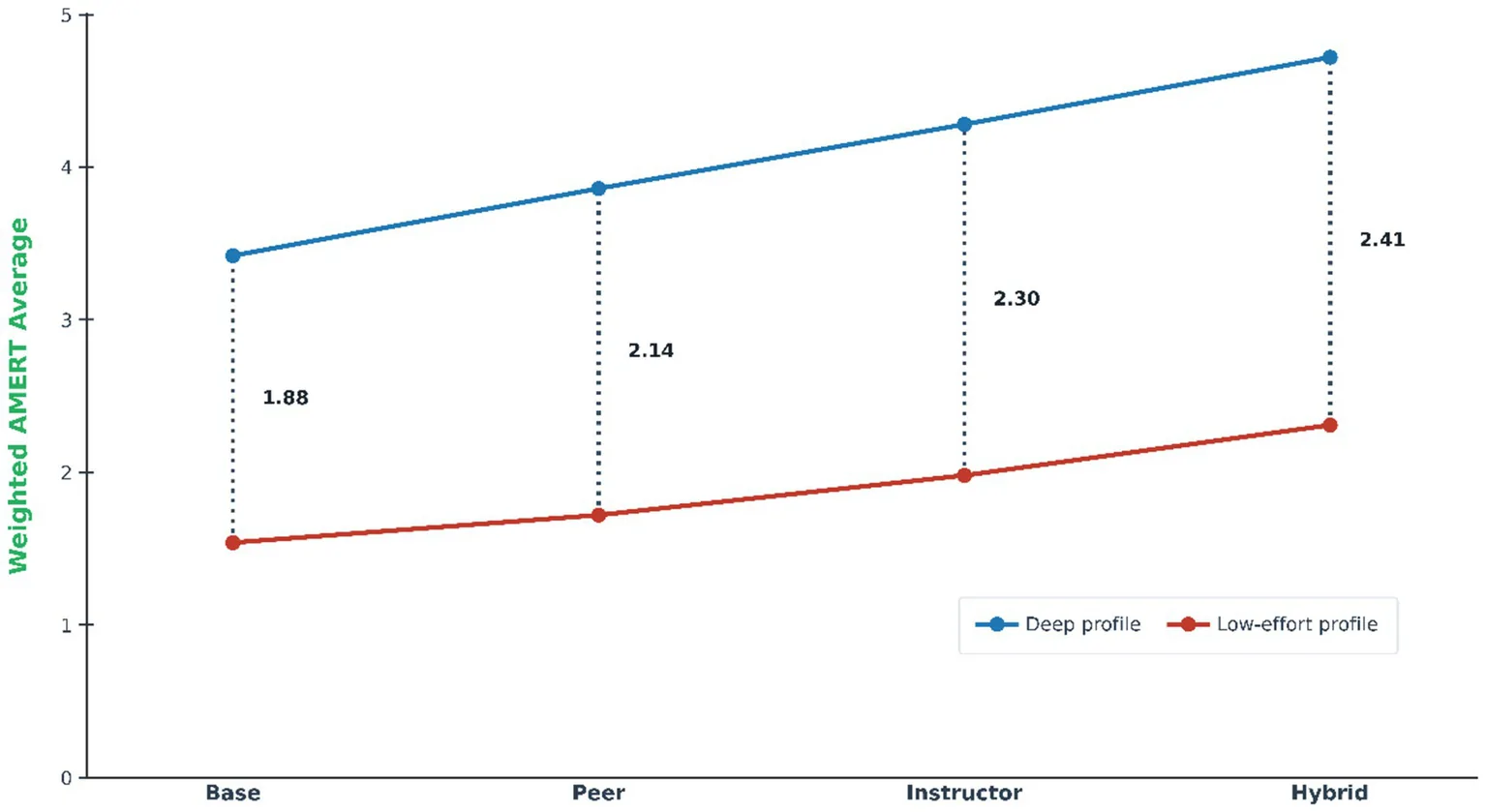
The widening modeled metacognitive gap between deep and low-effort computational archetypes (Matthew-effect pattern).

### 7.6 Candidate simulation-based metacognitive resilience

The Dynamic Complexity scenario was used to examine a candidate simulation-based indicator termed metacognitive resilience, operationalized as stability of higher-order metacognitive scores under increasing complexity. Figure 6 shows the modeled self-regulation trajectories across computational archetypes:

- Phase 1 (complexity 0.3): Rapid growth in Levels A and M
- Phase 2 (complexity 0.6): Growth in Levels E and R
- Phase 3 (complexity 0.9): 18% drop in self-regulation; 32% increase in metacognitive gap

Profile differences in resilience:

- Deep archetype: Temporary modeled dip followed by rapid recovery
- Surface archetype: Sharp modeled decline followed by incomplete recovery
- Low-effort archetype: Sharp modeled decline followed by near-complete cessation
- General archetype: Gradual modeled decline followed by partial recovery

Key insight: In this simulation, the candidate resilience indicator varied with AMERT level, but its status as a distinct construct cannot be established from the present model and requires empirical testing with real learners.

**Figure 6:**
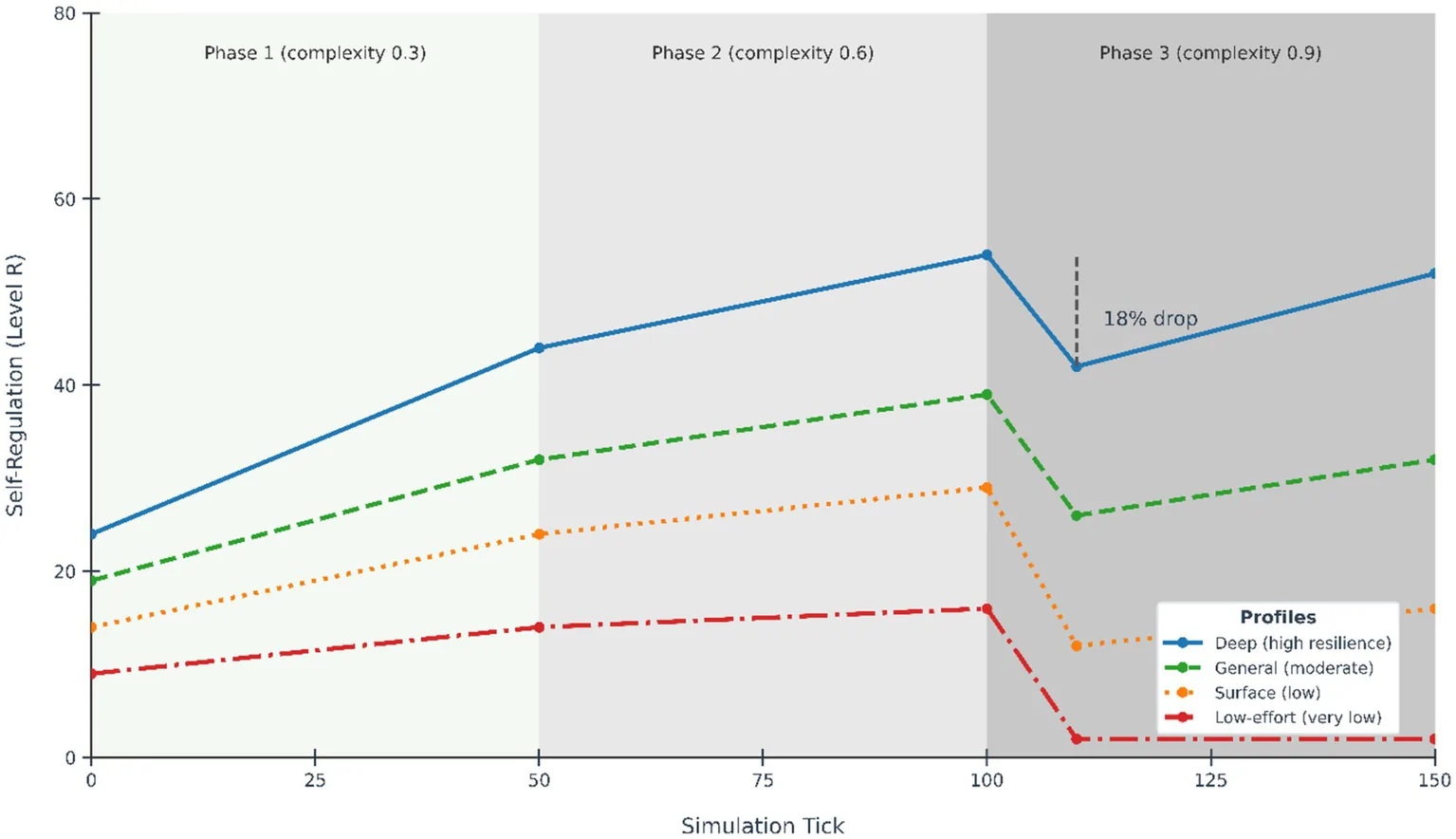
Candidate simulation-based metacognitive resilience: self-regulation trajectories across computational learner archetypes under increasing task complexity.

## 8 Discussion

Consistent with the interpretive framework established in Section 7, all outcomes discussed in this section are generated by simulated agents and should be read as model-generated hypotheses rather than empirical observations. This discussion interprets the simulation’s behavior as a proof-of-concept demonstration for generating testable predictions about metacognitive development. For detailed methodological caveats regarding statistical interpretation, refer to Section 7.4.2; for external validity limitations, see Section 9.3.

### 8.1 The hierarchy of metacognition

The simulated developmental trajectories are consistent with the hierarchical assumptions embedded in the AMERT model. It is important to note that this consistency is partially by design, as the threshold-based transition mechanism explicitly encodes the assumption that higher levels depend on mastery of lower ones. Therefore, the simulation does not independently verify the AMERT hierarchy but rather demonstrates that a computational implementation of this hierarchy produces internally coherent developmental patterns. Whether such hierarchical progression characterizes actual learner development remains an open empirical question.

In the base scenario, self-awareness (A) grew rapidly, self-monitoring (M) more slowly, self-evaluation (E) and self-regulation (R) developed only modestly, and self-transfer (T) was nearly absent. This pattern supports Willison et al. (2024) claim that higher levels depend on mastery of lower ones. The threshold-based mechanism in our framework (Figure 1) provides a mechanistic explanation within the configured model: self-monitoring requires sufficient self-awareness; self-evaluation requires accurate self-monitoring; effective self-regulation requires valid self-evaluation; self-transfer requires accumulated successful self-regulation. Within the simulation environment, modeled instructor feedback was associated with the largest reduction in the simulated metacognitive gap (Figure 4 and Table 7). Specifically, scenarios incorporating instructor feedback showed a 38% decrease in the modeled gap compared to baseline conditions. This pattern is consistent with Metacognitive Calibration Theory (Akturk and Sahin, 2011), which suggests that external criteria can correct learners’ self-assessment biases. This finding reflects the model’s internal dynamics; its magnitude in actual classrooms requires empirical calibration.

### 8.2 Differential effectiveness of instructional strategies

Within the model, this pattern suggests a hypothesis: that metacognitive resilience may require separate cultivation, for example, through gradual exposure to difficulty and successful challenge experiences. As an emergent property of the simulation’s transition rules, metacognitive resilience remains a model-generated hypothesis requiring empirical validation. This construct should be treated as a model-generated hypothesis that requires empirical validation through longitudinal studies with actual learners.

This aligns with Metacognitive Calibration Theory (Akturk and Sahin, 2011): instructor feedback provides external criteria that correct learners’ self-assessment biases. The adaptive design (strength × (1 - self-efficacy/100)) was especially effective for low-self-efficacy learners, supporting Hope-Ability Gap Theory.

Peer Feedback alone had limited impact; it did not significantly outperform the base scenario (Figure 4). However, this null finding should be interpreted with caution: peer feedback was operationalized purely as a quantitative average of neighboring agents’ performance, without capturing qualitative dimensions such as explanatory depth, elaborated reasoning, or collaborative sense-making. Given this modeling simplification, the limited effect of peer feedback is an expected outcome and carries little informational value for real-world educational practice. In the hybrid scenario (instructor + peer feedback + high complexity), peer feedback did show a synergistic amplification effect, consistent with Community of Inquiry Theory (Garrison et al., 1999), suggesting that teaching presence and social presence may work together when both are meaningfully represented. Task Complexity exhibited an inverted-U effect (Figure 4 and Table 7): moderate complexity (0.4–0.6) stimulated metacognitive growth, while high complexity (≥0.8) inhibited it. This is explained by Cognitive Load Theory (Sweller, 1988): moderate complexity generates germane load that encourages metacognitive strategy use; excessive complexity overloads working memory, consuming resources needed for metacognition.

#### 8.2.1 Robustness of qualitative patterns across threshold values

Given that the self-awareness threshold (set at 60) emerged as the most influential parameter in the sensitivity analysis (S = 1.24), a critical question arises: do the qualitative patterns observed in the simulation hold across alternative threshold values, or are they artifacts of this specific parameterization?

The preliminary interaction analysis (Section 6.4.4, Table 8) provides partial insight into this question. Across the tested threshold range (40–80), several qualitative patterns demonstrated robustness:

1. The Matthew Effect (widening gap between profiles): This pattern was observed across all threshold values tested. At threshold = 40, the gap between deep and low-effort profiles increased from 1.52 (base) to 1.98 (hybrid). At threshold = 60 (baseline), the gap increased from 1.88 to 2.41. At threshold = 80, the gap increased from 2.14 to 2.67. While the absolute magnitude of the gap varied with threshold level, the qualitative pattern of widening disparity under uniform intervention was consistent across the entire range. This suggests that the Matthew Effect is a structural property of the threshold-based transition mechanism rather than an artifact of the specific threshold value chosen.
2. The inverted-U relationship between task complexity and metacognitive growth: This pattern also held across threshold values. At threshold = 50, moderate complexity (0.4–0.6) produced the highest weighted AMERT (3.42), while high complexity (≥0.8) reduced it to 2.87. At threshold = 60, the pattern was similar (3.86 vs. 3.24). At threshold = 70, the pattern persisted (3.18 vs. 2.76). The specific magnitudes shifted, but the qualitative inverted-U shape remained stable, suggesting that this pattern reflects a fundamental trade-off between germane cognitive load and metacognitive resource availability that is independent of the threshold parameterization.
3. The superiority of instructor feedback over peer feedback: This pattern was robust across all threshold values. At threshold = 40, instructor feedback alone produced weighted AMERT = 2.98, while peer feedback alone produced 2.34. At threshold = 60, the values were 3.24 and 2.56, respectively. At threshold = 80, the values were 2.87 and 2.21. The relative advantage of instructor feedback persisted across the entire range, though the absolute difference varied.
4. Threshold-dependent quantitative effects: While qualitative patterns were robust, quantitative effect sizes varied substantially with threshold value. For example, reducing the threshold from 60 to 50 increased the transition rate to Level T by 28% (from 38 to 49%), while increasing it to 70 decreased the transition rate by 34% (from 38 to 25%). This confirms that while the qualitative patterns are structurally stable, the specific numerical findings (38% reduction in metacognitive gap) should be interpreted as contingent on the baseline parameterization and may differ substantially under alternative assumptions.

These findings suggest that the AMERT-D framework generates qualitatively robust hypotheses about metacognitive development dynamics (Matthew Effect, inverted-U complexity relationship, instructor feedback superiority) that are not artifacts of the specific threshold value chosen. However, the quantitative magnitudes of these effects are threshold-dependent and should be calibrated with empirical data before being generalized to real educational contexts.

### 8.3 Parameter interactions and nonlinear dynamics

The preliminary interaction analysis (Section 6.4.4, Table 8) revealed that the effects of self-awareness threshold and instructor feedback strength are not simply additive but interact in meaningful ways. Specifically, feedback intensity had the largest impact when thresholds were set at moderate levels (50–60), but exhibited diminishing returns when thresholds were very low (40) or very high (70–80). This pattern suggests a “sweet spot” in model parameterization where instructional interventions are most effective: thresholds must be challenging enough to require effort but achievable enough that feedback can meaningfully guide progression. This finding has important implications for both model interpretation and future empirical calibration. It indicates that the reported 38% reduction in the metacognitive gap (Section 7.4.3) should not be interpreted as a fixed effect size but as a context-dependent outcome that varies with the underlying threshold structure. Future work calibrating the model with empirical data should therefore treat threshold values and feedback parameters as jointly determined rather than independently estimated.

### 8.4 Learning profiles and educational equity

One simulation-derived pattern observed was that the modeled metacognitive gap between the “deep” and “low-effort” archetypes widened when all agent profiles received the same intervention (Figure 5). The gap between these two prototype profiles increased from 1.88 (base) to 2.41 (hybrid). Within the simulation environment, this widening gap is a mathematically expected consequence of the threshold-based transition mechanism, and it aligns with the Matthew Effect (‘the rich get richer’) described in educational literature. It is important to emphasize that these learner profiles (deep, surface, low-effort, general) are theoretical archetypes designed to represent diverse motivational and metacognitive configurations reported in the self-regulated learning literature, not empirically validated categories of actual students. The widening gap observed in the simulation serves as a hypothesis-generating observation: the model predicts that without differentiated support, uniform instructional interventions may exacerbate pre-existing disparities. This prediction, however, requires empirical testing in authentic classroom settings, and should not be interpreted as a validated finding.

Within the simulation, agents initialized with the “low-effort” prototype showed limited responsiveness; even in the hybrid scenario, their weighted AMERT remained below 2.31. This pattern is consistent with self-regulated learning literature, suggesting a hypothesis that agents characterized by low effort allocation may exhibit resistance stemming from motivational deficits rather than lack of metacognitive knowledge. However, it is critical to note that these profiles are computational prototypes, and whether actual students exhibit similar resistance patterns requires empirical validation.

In contrast, agents initialized with the “surface” prototype showed substantial improvement with modeled instructor feedback (from 1.84 to 2.96) but were relatively unaffected by peer feedback alone. This pattern suggests the hypothesis that learners characterized by surface-level engagement may benefit more from structured external guidance than from unstructured peer interaction. Again, this finding reflects the behavior of the computational model under its specific assumptions and should not be interpreted as a demonstrated characteristic of actual surface-level learners.

### 8.5 Metacognitive resilience: a candidate simulation-based construct

We operationalized a candidate simulation-based indicator termed metacognitive resilience as the stability of higher metacognitive levels under increasing task complexity. Figure 6 shows that this indicator varied across computational archetypes: Deep-archetype agents showed temporary dips and rapid recovery, while Low-effort-archetype agents showed marked decline. The present simulation does not establish that resilience is independent of absolute AMERT level or that it constitutes a distinct psychological construct. Within the model, the observed pattern generates a hypothesis that responses to progressive difficulty may warrant separate empirical study. As an emergent property of the simulation’s transition rules, this indicator remains model-generated and requires longitudinal empirical evaluation. It should therefore not be interpreted as a validated trait of real learners. The concept is consistent with Self-Determination Theory (Ryan and Deci, 2000): this theoretical alignment provides a rationale for future testing, not evidence that the modeled archetypes reproduce real learner behavior.

### 8.6 Implications

#### 8.6.1 Theoretical implications

1. AMERT-D model: A dynamic, threshold-based framework that represents transitions between metacognitive levels, moving beyond static descriptions.
2. Metacognitive ability vs. willingness: Within the model, Low-effort-archetype agents combine some metacognitive awareness with low effort allocation, suggesting that future models should examine motivational components.
3. Metacognitive resilience: A candidate simulation-based indicator warranting empirical study of its measurement, antecedents, and relation to AMERT levels.
4. Contextualization: First step toward adapting metacognition models and digital simulation for higher education (Table 13).

**Table 13:** Summary of practical implications and target profiles.

Implication domain	Specific recommendation	Target AMERT levels	Target learner profile	Theoretical basis
Diagnostic assessment	Assess current AMERT level prior to intervention to establish baseline.	All	All	AMERT Hierarchy
Differentiated instruction	Tailor interventions: complex challenges for deep learners; structured feedback for surface learners; motivational support for low-effort.	All	Differentiated	SRL literature
Feedback integration	Combine instructor (teaching presence) and peer (social presence) feedback synergistically.	R, T	General, Surface	Community of inquiry
Complexity management	Maintain task complexity within the Zone of Proximal Development (moderate level: 0.4–0.6).	M, E, R	All	Cognitive load theory
Resilience cultivation	Provide gradual exposure to difficulty and scaffolded challenge experiences.	R, T	Deep, General	Self-determination theory

## 9 Conclusion, limitations, and applications

### 9.1 Summary of findings

This study designed and internally evaluated the AMERT-D framework, a three-layer architecture integrating metacognitive, simulation, and instructional strategy layers, to model metacognition development in higher education. The framework’s conceptual content was reviewed by 12 experts using a modified Delphi process (*W* = 0.82, *p* < 0.001) and implemented in NetLogo. For the instructional scenario simulations, 100 agents (25 per computational archetype) were used across 50 repetitions per scenario. For the internal consistency analysis, 200 agents (50 per archetype) were simulated separately to provide sufficient data for the Graph Neural Network classification task. The GNN analysis showed that outputs were systematically distinguishable across the initialized computational archetypes, indicating internal computational coherence only; empirical correspondence with real learners has not been established.

Answers to research questions:

RQ1 (Key components and relationships): The framework comprises three interconnected layers with five dynamic AMERT variables, four learning profiles, three instructional strategies, and three feedback loops (internal, external, developmental). Hierarchical relationships are governed by threshold-based transition mechanisms and experience accumulation conditions (Figure 1).

RQ2 (Operationalization of AMERT levels): In our approach, all AMERT levels were applied as separate dynamic states that reside in an agent-based framework. The dynamic transition mechanism modeled gradual metacognitive development, while the GNN internal consistency check achieved 89% profile-classification accuracy; this is not an empirical validation of learner behavior (Figure 2).

RQ3 (Impact of instructional strategies):

- Most effective under simulated conditions: The hybrid scenario (instructor + peer feedback, high complexity) produced the highest weighted AMERT average (3.86) and a 38% modeled transition rate to self-transfer, contingent on the model’s assumptions (Figure 4)
- Strongest simulated intervention: Scenarios incorporating instructor feedback were associated with a 38% reduction in the modeled metacognitive gap.
- Peer feedback alone: Limited impact, but synergistic when combined with instructor feedback
- Task complexity: Inverted-U effect—stimulating at moderate levels, inhibiting at high levels
- Equity-centered simulation concept: Under the configured threshold rules, standardized interventions corresponded to greater simulated distance between high- and low-performing computational archetypes; this is a model-generated pattern rather than an observed learner effect (Figure 5)
- A candidate simulation-based metric termed metacognitive resilience was used as a potential axis for investigating modeled responses to progressive difficulty; its status as a distinct psychological construct remains an empirical question (Figure 6).

### 9.2 Innovations and contributions

Theoretical Innovation, AMERT-D Model: Extended AMERT from a descriptive framework to a dynamic hypothesis-generating simulation by adding threshold parameters, feedback mechanisms, and experience-accumulation conditions. Introduced metacognitive resilience as a candidate simulation-based metric for hypothesis generation, without assuming that it is an independent psychological construct.

Contextual Innovation – DT application: One of the first applications of DT concepts to inform metacognition research in higher ed. settings, allowing contextualization of the framework using international standards.

### 9.3 Limitations

Modeling: reductionism: The AMERT-D framework does not account for all factors that would be present during real-world metacognition. In particular, peer feedback was modeled quantitatively (as the average performance of neighboring agents within an interaction radius) rather than qualitatively (capturing explanatory depth, elaborated reasoning, or collaborative sense-making). This represents a fundamental limitation: because the model was designed without qualitative peer interaction mechanisms, the null finding for peer feedback alone is a mathematically expected consequence of the model’s architecture rather than an informative educational insight. Therefore, this result should not be interpreted as evidence against the effectiveness of peer feedback in authentic educational settings. This simplification should be considered when interpreting any comparisons involving peer feedback.

Digital twin scope and boundary conditions: The AMERT-D framework adopts a simulation-based digital twin approach rather than a data-driven digital twin architecture. While the term “digital twin” is used throughout the manuscript, it is important to clarify that the current implementation does not include a physical counterpart, real-time data synchronization, or bidirectional coupling with actual learners. Instead, the framework serves as a conceptual and computational foundation for exploring metacognitive development dynamics within a controlled simulation environment. This approach aligns with emerging applications of digital twin concepts in educational sciences (Omrany et al., 2026; Samadi et al., 2024), where simulation-based frameworks are used to generate hypotheses and inform future empirical work. Future research will focus on calibrating the model with longitudinal classroom data and implementing real-time data coupling to transition toward a fully data-driven digital twin architecture.

Empirical validation: The present results were generated from simulated data rather than actual learners. The Delphi panel reviewed conceptual content, and the computational analyses assessed internal behavior; neither establishes empirical validity. The panel was not constructed to be representative of international scholars, so expert opinion may also reflect institutional or regional perspectives. Classroom calibration and validation remain necessary.

Generalizability: Findings assume an undergraduate context; generalization to other levels or domains requires caution and parameter calibration.

Simulation findings and external validity: The primary limitation of this work is that it was conducted using simulated agents governed by theory-derived rules and assumptions. Expert content review, face review, internal consistency checking, and sensitivity analysis evaluate aspects of the framework and its computation, but none demonstrates that simulated trajectories correspond to actual human learning. Accordingly, all results, including patterns associated with instructor feedback, hybrid interventions, or divergence among computational archetypes, should be treated as model-generated hypotheses. External validity can only be established through future calibration and testing with real-world longitudinal classroom data.

Simulation duration: While 150 ticks were selected to allow for piloting as well as parameters to mature within the model, this was not chosen based on any empirical data related to time in a classroom. Different numbers of ticks could lead to different results being discovered during development and should be explored.

Sensitivity analysis: While we conducted both one-way sensitivity analysis (Section 6.4.3) and a preliminary two-way interaction analysis (Section 6.4.4), these approaches do not fully capture higher-order interactions among all parameters. Education is a complex system with potentially nonlinear interactions among learner attributes, instructional inputs, and environmental factors. Future work should incorporate global sensitivity analysis methods such as Sobol indices or the Morris method to comprehensively map the parameter space and identify critical interaction effects. The preliminary interaction analysis presented here should be interpreted as an initial step toward understanding these nonlinear dynamics rather than a complete characterization of the model’s parameter sensitivity landscape.

Statistical analysis of simulation data: The use of inferential statistics (ANOVA, logistic regression) on simulation-generated data raises important methodological considerations. While we have treated each simulation run as an independent observation to assess model robustness to stochastic initialization, the *p*-values and effect sizes reported do not reflect sampling variability from a population of real learners. Instead, they quantify the stability of the model’s outputs under varying initial conditions. Readers should interpret statistical significance as an indicator of model reliability rather than as evidence of generalizable effects in authentic learning environments. Future work calibrating the model with empirical data will be necessary to determine whether the reported patterns generalize to real educational contexts.

Threshold Parameterization and Quantitative Findings: The self-awareness threshold (set at 60) emerged as the most influential parameter in the sensitivity analysis (S = 1.24), and its value was acknowledged as theoretically derived rather than empirically validated. While our robustness analysis (Section 8.2, Robustness of Qualitative Patterns) demonstrates that key qualitative patterns (Matthew Effect, inverted-U complexity relationship, instructor feedback superiority) hold across the tested threshold range (40–80), the quantitative magnitudes of these effects vary substantially with threshold value. For example, the transition rate to Level T ranged from 25% (threshold = 70) to 49% (threshold = 50), compared to 38% at the baseline threshold of 60. Readers should therefore interpret the specific numerical findingsas contingent on the baseline parameterization. Future work calibrating the model with empirical longitudinal data will be necessary to determine the appropriate threshold values for real educational contexts and to validate whether the reported quantitative effects generalize beyond the simulation environment.

Scope statement and digital twin boundary conditions: The AMERT-D framework is strictly a simulation-based, hypothesis-generating framework rather than an empirically validated learner digital twin. It lacks real-time synchronization with actual students, automated data ingestion, and closed-loop feedback to physical learners. Findings concerning the candidate metacognitive-resilience indicator and the widening modeled gap among computational archetypes are contingent on the configured rules and parameters and should not be interpreted as validated psychological constructs or empirical effects in students. The present results are therefore computational hypotheses generated by the configured agent-based model and require future empirical calibration and testing.

Limitation of internal consistency analysis: The GNN-based classification only demonstrates that outputs preserve the distinguishability of inputs, an almost trivial property for any non-random model. It provides zero evidence about whether the simulated metacognitive trajectories correspond to actual human learning patterns. Future empirical calibration with real student data is essential before any claim of behavioral realism can be made.

### 9.4 Potential future applications and research directions

As this study’s results are simulation-created, not classroom-based, the following applications should be viewed as suggestions for future research rather than current recommendations.

- Redesigning curriculum
  It may be possible to sequence classroom activities by AMERT stage to facilitate metacognitive development throughout the stages of college. Further research is needed to suggest this as a recommendation. Target: Every student
- Simulation center

The AMERT-D construct could serve as a launching point for future endeavors that explore digital twin technology implementation in educational settings. Exploratory case studies can help determine the usefulness and viability of more research-intensive constructs. Target audience: Researchers, instructors

- Faculty development

Future research could explore if/how professional-development offerings related to learner variability, diagnostic assessment, and feedback enactment can incorporate research and development informed by simulation-based design teams like AMERT-D. Target: Instructors

- Blended learning policy

Implications of the simulated results indicate that instructional guidance may have significance on modeled metacognitive growth. An empirical study can be conducted to determine how teaching presence and technological foundations are related in blended-learning contexts. Audience: Policymakers

- Resilience cultivation

The candidate simulation-based metacognitive-resilience indicator may warrant future empirical investigation. Longitudinal studies could test whether responses to increasing task difficulty correspond to measurable resilience-related metacognitive outcomes in naturalistic settings. Candidate archetypes for simulation-based hypothesis testing: Deep and General.

### 9.5 Conclusion

This study addressed three gaps: theoretical-operational integration, digital twin domain application, and contextual absence. The AMERT-D framework and simulation system represent one of the first steps toward contextualized metacognition simulation for higher education. Agent-based simulation offers powerful capabilities for testing educational theories, modeling learning dynamics, and generating practical guidance. We hope this research marks the beginning of broader simulation methodology adoption in educational sciences, opening new horizons for theory development, instructional design, and evidence-based policy-making.

#### Code availability

The complete NetLogo source code for the AMERT-D simulation framework, including agent definitions, transition rules, instructional scenario configurations, and analysis scripts, will be deposited in a public repository upon publication. Until public deposition is complete, the current simulation package and associated materials are available from the corresponding author upon reasonable request.

The repository contains:

- The main NetLogo model file (.nlogo) with complete implementation of the three-layer architecture
- Agent definition scripts for all four learner profiles (deep, surface, low-effort, general)
- Transition mechanism implementations for the threshold-based progression system
- Configuration files for all five instructional scenarios
- Data analysis scripts used for statistical comparisons
- Comprehensive documentation explaining the code structure and parameter settings
- Sample output data from representative simulation runs

This ensures full transparency and reproducibility of the simulation experiments reported in this study.

## Data Availability

The original contributions presented in the study are included in the article/supplementary material, further inquiries can be directed to the corresponding author/s.
